# Nurse Staffing and Nursing Home Deficiency of Care for Inappropriate Psychotropics Use in Residents With Dementia

**DOI:** 10.1093/geroni/igaa057.671

**Published:** 2020-12-16

**Authors:** Jung Min Yoon, Alison Trinkoff, Carla Storr, Elizabeth Galik

**Affiliations:** 1 University of Maryland, Baltimore, Baltimore, Maryland, United States; 2 University of Maryland, Baltimore, Maryland, United States

## Abstract

Psychotropics use to manage behavioral and psychological symptoms of dementia (BPSD) in nursing homes (NHs) has been the focus of policy attention due to their adverse effects. We hypothesized that NHs with lower nursing staffing would have greater reliance on psychotropics use to control BPSD. A NH deficiency of care can be cited for inappropriate psychotropics use (F-tag 758). The association between the occurrence of F-758 tags and nurse staffing in residents with dementia was examined using the 2017-18 Certification and Survey Provider Enhanced Reporting data (n=14,548 NHs). Staffing measures included nursing hours per resident day (HPRD) and registered nurse (RN) skill-mix. Generalized linear mixed models that included covariates (NH location, bed size, ownership, proportion of residents with dementia/depression/psychiatric disorders and with Medicare/Medicaid) estimated the magnitude of the associations. There were 1,872 NHs with F-758 tags indicating inappropriate psychotropics use for NH residents with dementia. NHs with greater RN and certified nurse assistant (CNA) HPRD had significantly lower odds of F-758 tags (OR=0.59 54, 95% CI=0.47 44-0.73 66; OR=0.87, 95% CI=0.77-0.99, respectively) and similar findings were found in NHs with greater RN skill-mix (OR=0.14 10, 95% CI=0.05 04-0.37 25). There were no significant associations between the occurrence of F-758 tags and licensed practice nurse and unlicensed nurse aide HPRD. This study found that RN and CNA staffing had inverse associations with inappropriate psychotropic use citations among residents with dementia. NHs with higher RN staffing ratios may be better able to implement alternatives to pharmacological approaches for BPSD. It is suggested that NHs be equipped with adequate nurse staffing levels to reduce unnecessary psychotropics use.

